# A commentary on ‘Assessment of treatment outcomes: cytoreductive surgery compared to radiotherapy in oligometastatic prostate cancer – an in-depth quantitative evaluation and retrospective cohort analysis’

**DOI:** 10.1097/JS9.0000000000001446

**Published:** 2024-04-09

**Authors:** Jiacheng Chen, Jincai Wu, Haijing Yu

**Affiliations:** a Department of Hepatobiliary and Pancreatic Surgery, Hainan General Hospital (Hainan Affiliated Hospital of Hainan Medical University); b Department of International Nursing School, Hainan Medical University, Haikou, Hainan Province, People’s Republic of China

*Dear Editor*,

We have read with great interest the study conducted by Cheng *et al*.^1^, which investigated the efficacy of cytoreductive surgery compared to radiotherapy in managing patients with oligometastatic prostate cancer. In this study, the authors performed a pooled analysis comprising five studies involving 1425 patients and demonstrated that cytoreductive surgery was associated with significantly improved cancer-specific survival and overall survival when compared to radiotherapy. However, there were no significant differences observed between the two groups in terms of progression-free survival (PFS) and castration-resistant prostate cancer-free survival (CRPCFS). The authors should be commended for providing up-to-date evidence supporting the superiority of cytoreductive surgery in oligometastatic prostate cancer management. Nevertheless, there are several concerns that require further clarification prior to the translation of these findings into clinical practice.

First, accurate data extraction is a fundamental prerequisite to ensure the reliability of meta-analysis results. Among the included studies, Patel *et al*.’s study^2^ clearly reported both univariate and multivariate overall survival and cancer-specific survival outcomes between the two groups in its supplementary file. However, the data provided by this meta-analysis were pretty inconsistent with those of the primary study. Furthermore, upon careful examination of the original article, we discovered that Patel *et al*.^2^ did not directly or indirectly report PFS and CRPCFS. Further clarification is needed regarding whether the data included in this meta-analysis were obtained through communication with Patel and colleagues. Additionally, Cheng and colleagues erroneously extracted the sample size for cytoreductive surgery from Patel *et al*.’s study^2^ (176 patients are for radical prostatectomy±external beam radiation therapy, not for radical prostatectomy alone). Equally concerning, we found that Xue *et al*.’s study^3^ did not report PFS between the two groups; it remains unclear how these authors extracted this particular data.

Second, as a well-established statistical concept, the hazard ratio cannot be less than zero. However, we found that the statistical results of several forest plots in the present study were negative, such as Figures 2–1, 4, 5–1, and 5–2. The methodology employed in generating these forest plots remains perplexing.

Last, the keyword ‘radiotherapy’ in the text is misspelled as ‘rediotherapy’, which necessitates further verification by the authors to determine if this will result in invalid retrieval.

## Ethical approval

No need for this letter to the editor.

## Sources of funding

This study was supported by Natural Science Foundation of Hainan Province (No. 822QN320).

## Author contribution

C.J.C. and W.J.C.: original draft conception and writing; Y.H.J.: critical revision of the manuscript. All authors reviewed the manuscript.

## Conflicts of interest disclosure

The authors have no competing interests to declare.

## Research registration unique identifying number (UIN)

1. Name of the registry: none.
2. Unique identifying number or registration ID: none.
3. Hyperlink to your specific registration (must be publicly accessible and will be checked): none.

## Guarantor

Haijing Yu.

## Provenance and peer review

Commentary, internally reviewed.
